# Sleep Extension Normalizes ERP of Waking Auditory Sensory Gating in Healthy Habitually Short Sleeping Individuals

**DOI:** 10.1371/journal.pone.0059007

**Published:** 2013-03-08

**Authors:** Valentina Gumenyuk, Oleg Korzyukov, Thomas Roth, Susan M. Bowyer, Christopher L. Drake

**Affiliations:** 1 Henry Ford Hospital, Sleep Disorders and Research Center, Detroit, Michigan, United States of America; 2 Department of Communication Sciences and Disorders, Northwestern University, Evanston, Illionois, United States of America; 3 Henry Ford Hospital, Neuromagnetism laboratory, Neurology Department, Detroit, Michigan, United States of America; 4 Wayne State University, Detroit, Michigan, United States of America; University of Pennsylvania School of Medicine, United States of America

## Abstract

Chronic sleep loss has been associated with increased daytime sleepiness, as well as impairments in memory and attentional processes. In the present study, we evaluated the neuronal changes of a pre-attentive process of wake auditory sensory gating, measured by brain event-related potential (ERP) – P50 in eight normal sleepers (NS) (habitual total sleep time (TST) 7 h 32 m) vs. eight chronic short sleeping individuals (SS) (habitual TST ≤6 h). To evaluate the effect of sleep extension on sensory gating, the extended sleep condition was performed in chronic short sleeping individuals. Thus, one week of time in bed (6 h 11 m) corresponding to habitual short sleep (hSS), and one week of extended time (∼ 8 h 25 m) in bed corresponding to extended sleep (eSS), were counterbalanced in the SS group. The gating ERP assessment was performed on the last day after each sleep condition week (normal sleep and habitual short and extended sleep), and was separated by one week with habitual total sleep time and monitored by a sleep diary. We found that amplitude of gating was lower in SS group compared to that in NS group (0.3 µV *vs*. 1.2 µV, at Cz electrode respectively). The results of the group × laterality interaction showed that the reduction of gating amplitude in the SS group was due to lower amplitude over the left hemisphere and central-midline sites relative to that in the NS group. After sleep extension the amplitude of gating increased in chronic short sleeping individuals relative to their habitual short sleep condition. The sleep condition × frontality interaction analysis confirmed that sleep extension significantly increased the amplitude of gating over frontal and central brain areas compared to parietal brain areas.

## Introduction

Chronic sleep loss has been associated with increased daytime sleepiness, as well as impairments in memory and attentional processes [1]–[3]. Nevertheless, chronic short sleep despite a need for more sleep (regularly sleeping ≤6 h) is an increasingly common condition in modern society due to professional demands, social and technological availability, and domestic responsibilities [4].

A previous study looking at the cumulative effect of chronic sleep restriction on changes in measures of sleepiness and neurobehavioral performance found that the first two days of restricted sleep (5–6 h time in bed [TIB]) increased sleepiness significantly relative to 7–8 h TIB, and performance decrements in reaction time showed significant changes after the second day of sleep restriction. The deficits continued to grow throughout the final day of the sleep restriction study, albeit in a negatively accelerating fashion [5]. In habitual short sleepers (sleep episode <6 h), the evidence of higher homeostatic sleep pressure for “non-REM sleep” was demonstrated [6]. All these findings suggest that *cumulative sleep loss* produces a decrement in behavioral performance and alertness and is likely to be experienced by people who are habitual short sleepers in the general population [7]. The neuronal mechanism of these processes is not yet known.

There are a number of electrophysiological and imaging studies that address the changes in brain activity associated with total and partial sleep deprivation. In an electrophysiological study evaluating the effect of sleep restriction from 8.29 h to 5.38 h TIB on measures of waking arousal (defined by EEG power of theta and low/high alpha frequencies), a reduction of power of waking EEG on the frontal sites was observed after the first night of sleep restriction, and subsequent linear effects of sleep loss on EEG power at central and parietal brain regions were observed in healthy subjects for the following 7 days [2]. In our previously published study [3], we demonstrated that habitual short sleep (<6 h) is associated with a deficit specifically in the frontal neuronal mechanism of auditory novelty processing, evaluated by the fontal-central distributed and attention-dependent event-related brain potential (ERP) component (P3a) in healthy volunteers.

In a sleep deprivation study, it was found that 40 hours of sleep deprivation produced an increase in power density of delta and theta activity over frontal EEG derivations relative to parietal and occipital areas in young healthy subjects [8]. An ERP study done by Gosselin et al. [9] demonstrated the effects of total sleep deprivation on novelty processing in an attended (subjects actively attended to the sounds) sound stream. Their finding showed a significant reduction of P3a amplitude after total sleep deprivation, specifically over frontal brain areas, in healthy participants. A positron emission tomography study by Thomas et al. [10] demonstrated that 24 hours of sleep deprivation produces global decreases in activity across many brain areas, with larger reductions in the activity of the thalamus, prefrontal, and posterior parietal cortices. It is important to note that these areas of the brain are involved in attention and memory functions. Furthermore, a study done by Drummond and colleagues [11] demonstrated the impact of sleep deprivation on both psychomotor performance and on the “default mode network,” which consists of the frontal and posterior brain regions essential for attention. Based on the relatively large number of sleep deprivation studies and sleep restriction studies looking at neuronal network changes, there is clear evidence supporting the impact of sleep loss on broad areas of the brain, but primarily on frontal areas, which affect waking functions, particularly the domain of attention. Studies addressing the impact of sleep loss on neuronal activity related to the domain of attention are necessary due to the unclear elucidation of the neurophysiological mechanisms of functional deficits caused by sleep restriction.

In the present study, we evaluated the neuronal changes of a pre-attentive process which does not rely on participant's motivation and behavioral performance, but involves activation of the brain regions essential for inhibition and attention [12] which are negatively impacted by sleep loss. The filtering out of redundant sensory information is a neurophysiological process operated pre-attentively (automatically), involving subcortical and cortical brain structures. This process is referred to as “gating” and is a fundamental brain function – it prevents our sensory neuronal system from becoming overloaded by irrelevant information and thus enables the brain to perform higher order cognitive processes [13]. In the auditory modality, the process of gating has been assessed in a number of studies utilizing the P50 auditory event-related potential (ERP), recorded from the vertex at a latency of ∼50 ms in response to a pair of click-stimuli (termed S1 and S2). The P50 is a complex consisting of two subsequent components: Pb1 (peaking ∼50 ms) and Pb2 (peaking ∼70 ms from stimulus onset) (Yvert et al., 2001). The amplitude ratio between the P50 response to the S1 stimulus and the P50 response to the S2 stimulus is an electrophysiological marker of sensory gating. The amplitude difference between S1 (conditioning stimulus) and S2 (testing stimulus, [14]) indicates the “sensory gating” process: the less the difference, the lower the gating. In normal healthy subjects, it was found that during wakefulness, the P50 elicited by S2 is decreased in amplitude compared to the P50 elicited by S1; this is due to inhibitory processes that act on redundant information [14], [15]. A gating deficiency may be a result of deficits in subcotrical and cortical neuronal inhibition and may lead to over-stimulation of the attentional network [14].

The deficit of gating has been largely observed in clinical studies involving psychiatric [16], [17] and neurological [18]–[20] patients. Importantly, these psychiatric and neurological disorders are often marked by comorbid sleep disorders. In patients with a sleep disorder (e.g., insomnia), P50-gating also has been shown to exhibit amplitude reduction as compared to normal healthy subjects [21], [22]. While it has been shown that sensory gating can be used for evaluation of the neuronal changes associated with the dysregulation of the sleep–wake cycle and attentional disturbances in neurological patients [20], [23], [24], at this time there are no studies demonstrating the association between reduction of sleep time and possible changes in the characteristics (i.e., amplitude and latency) of pre-attentive sensory gating in healthy subjects.

Studies examining the neuronal source underlying gating found that neuronal activation associated with this neurophysiological process is primarily mediated by the prefrontal and auditory cortices, with additional potential contributions from the thalamus (intracranial ERP [25] and fMRI [26]). Earlier studies demonstrated that at least one of the generators of the P50 is located in the pedunculopontine nucleus, which is one of the main components of the ascending reticular-activating system (RAS) [27], [28]. Thus changes in the characteristics of gating might indicate disturbances in arousal and sleep-wake regulation by the RAS, which is known to be active during wake and rapid eye movement (REM) sleep and less active during slow-wave sleep [29].

Finally, the relation between reduced vigilance and reduced gating is supported by studies showing suppression of gating amplitude by sedative drugs. Specifically, the cholinergic antagonist scopolamine [27], and exogenous melatonin [30] have both been shown to reduce gating amplitude in normal healthy subjects. On the other hand, a stimulant, such as the adenosine antagonist theophylline, significantly increased the P50 amplitude ratio between both S2 and S1 in healthy subjects [31]. A separate study with caffeine demonstrated correlation between dose of caffeine and P50 sensory gating in healthy subjects: subjects who consumed a lower dose of caffeine showed a lower amplitude P50 in response to S2 [32].

In the current study, we hypothesized that healthy individuals with habitual short total sleep time (<6 h), but without complaints of excessive sleepiness or insomnia, will nonetheless exhibit a reduction in auditory sensory gating amplitude (expressed by gating difference wave (GDW)) relative to that of normal sleeping subjects (7–8 h habitual sleep time). It was also hypothesized that one week of extended time in bed (∼8.4 h) would increase their waking auditory sensory gating comparable to that of healthy normal sleeping subjects. Thus the primary endpoint of this study was the gating responses as measured from difference waves (Gating Difference Wave or GDW) obtained by subtracting the averaged waveform to S2 stimuli from the averaged waveform to S1 stimuli.

## Methods

### Participants

All subjects reported no history of sleep disorders or sleep-wake complaints, and were working or attending school on a typical daytime schedule (∼0800–1700 hours, Monday – Friday). Ten healthy self-reported habitual short sleepers (SS) and 9 healthy self-reported normal sleepers (NS) were recruited from Henry Ford Health System to participate in our study. SS were individuals whose average total sleep time (TST) on a self-reported two-week sleep diary was 5.0–6.0 hours, and who also had a bedtime between 2100 and 0100, a time to sleep onset of less than 30 minutes, and a wake time between 0600 and 0900. Individuals who responded to our advertisement as self-reported SS, but did not fulfill these criteria, were disqualified from the study. NS are defined as individuals whose average TST on a self-reported two-week sleep diary was 7–8 hours/night. None of the participants reported insomnia or excessive sleepiness as determined by clinical interview and Epworth sleepiness Scale (ESS) scores (less than 10) as well as an Insomnia Severity Index scores (less than 10). Thus, despite being asymptomatic for excessive sleepiness, our habitual short sleeping individuals obtained less than the recommended 7–8 hours of habitual sleep per night which is necessary to prevent cumulative deterioration in performance in a variety of cognitive tasks [33].

The ERP data of two participants from the SS group and one participant from the NS group were contaminated by massive blink and movement artifacts and were therefore excluded from analysis. Thus, ERP and sleep data of eight short sleepers (age: [mean ± SD] 38.3 yrs ±11.5 yrs, 6 F) and 8 normal sleeping individuals (age: 29.5 yrs ±13.1 yrs, 5 F) are presented. All subjects were right handed, with normal hearing and vision, and free of psychiatric and neurological conditions. All participants were required to pass a health screening, which included a physical examination and an interview with a clinical psychologist specialized in sleep medicine. Participants were also required to score accordingly on the following questionnaires: Epworth Sleepiness Scale (ESS) less than 10 [34], Insomnia severity Index (ISI) less than 10 [35], no indication of sleep apnea on the Berlin questionnaire (each participant endorsing less than 2 categories on the Berlin indicating low risk for obstructive sleep apnea (OSA) [36], and healthy mental state on the Profile of Mood States questionnaire (POMS) [37]. All subjects were free of medications for a minimum of 2 weeks prior to study participation. Study entry criteria included non-smokers and caffeine use ≤300 mg/day.

The study was approved by the IRB committee of the Henry Ford Health System. Prior to taking part in the study, all participants were informed about the study and provided written consent. Participants were compensated for their participation.

### Groups

Participants were not significantly different for age (*t = 1.4; p<0.24*). They were assigned by their self-reported average total sleep time on a two-week sleep diary to each group as NS or SS. Habitual total sleep time was confirmed during the physical evaluation. Additional inclusion criteria are noted above (see participants).

### Sleep Conditions

The sleep conditions (habitual and extended) were counterbalanced in habitually short sleeping participants and performed for one week prior to the EEG/ERP assessment. A week of habitual time in bed was required between the 2 sleep conditions in order to “wash out” the effect produced by extended time in bed for each short sleeper, and monitored using a sleep diary. In the extension condition, the TIB was determined as follows: participants were asked to go to bed earlier by 1 h from their average 2-week diary bedtime and delay their habitual wakeup time by 1 h. The ERP assessment was performed on the last day after each sleep condition week (normal sleep and habitual short and extended sleep).

### Event-related potential recording procedure and analysis

The ERP recording session was performed between 14∶00 and 16∶00 hours. The ERPs were recorded using a 64 EEG-channel cap (10–20 system, Easy Cap, Gilching, Germany) and an ASA-EEG system (ANT, Netherlands). All participants were pre-screened with a brief hearing test using standard procedures prior to the ERP study. The two forehead electrodes (Fp1 and Fp2) as well as one left (F7) and one right (F8) served to monitor eye movement artifact during EEG recordings. Impedance was kept <10 kΩ. A band-pass filter was set from 0.1 to 100 Hz, and the sampling rate was 1024 Hz.

### Stimuli

During a recording session, 100 pairs of two identical tones (clicks) (S1 and S2; sinusoidal waves, frequency 1500 Hz, Gaussian envelope, duration 4 ms, onset and decay phase of 1.2 ms each) were presented (intensity 75dB) binaurally via headphones with an interstimulus interval of 500 ms and an interpair interval of 8 sec [38]. The VAPP system (http://nrc-iol.org/vapp/) was used for generating and presenting auditory stimuli.

All participants were seated comfortably in a sound-attenuated room in the sleep center, and asked to ignore all presented sounds. A silent movie, chosen by the participants, was used to help subjects follow task instructions to “ignore the auditory stimuli and to direct their attention to the movie with subtitles.”

EEG data were analyzed using Brain Vision Analyzer software (Brain Products GmbH, Gilching, Germany). ERP data were segmented separately for stimulus 1 and stimulus 2, starting with 100 ms prior to stimulus onset and continuing for 70 ms after the stimulus onset. A band-pass filter ranging from 1 to 50 Hz was applied to segmented data. Trials in which the EEG exceeded ±70 μV were excluded from the analysis. ERPs in response to S1 and S2 stimuli were averaged separately. On average, more than 80 trials for each click were included for each individual's grand average. Baseline correction (100 ms pre-stimulus interval) was applied to the averaged data.

The P50 response was measured from the averaged waveform to the S1 (first) stimulus of the pair as the largest positive polarity peak within the range of 40–60 ms from stimulus onset at the Cz electrode for each subject. To evaluate the potential differences across subjects in P50 peak amplitude to the first stimulus, the peak amplitude of the P50 response was compared between and within groups by ANCOVA, where age was used as a covariate factor.

The gating responses were measured from difference waves (Gating Difference Wave or GDW) obtained by subtracting the averaged waveform to S2 stimuli from the averaged waveform to S1 stimuli. The GDW was computed by point-to-point subtraction of the S2 waveform from the S1 waveform. We used the GDW to isolate a portion of the brain activity that is elicited by the presentation of the second stimulus in the pair, and to distinguish the process of gating from processes underlying the P50 generation [25], [26]. The lower amplitude of the GDW indicates a poor gating process. *3-way ANOVAS* were used for comparison of the GDW difference (mean amplitude across the 10 ms time window around the peak of GDW) between groups ([NS *vs*. hSS] and [NS vs. eSS], across frontal (F3, Fz, F4)/central (C3, Cz, and C4), and parietal (P3, Pz, and P4) [frontality] and left (F3, C3, and P3)/central (Fz, Cz, and Pz)/right (F4, C4, and P4 [laterality] locations). In a within-group (chronic short sleepers) analysis, *3-way ANOVAS* were used to evaluate differences of the GDW between sleep conditions [habitual vs. extended], across frontality and laterality locations. Age was included as a covariate in analysis.

In all statistical analyses using ANCOVA, Greenhouse-Geisser corrections were applied when appropriate. Significant main effects were further tested by Newman-Keuls post hoc testing.

Sleep diary data from each group was compared by *independent group t*-*tests*, whereas the sleep diary data corresponding to each sleep condition was compared *by dependent samples t-tests*; all results are presented in Table 1.

**Table 1:** One Week – Sleep Diary (Mean ± SD) Obtained from Normal Sleepers, Habitual Short Sleepers, and Extended Sleep Condition.

	Short Sleep	Normal Sleep	*P-value (SS vs. NS)*	Extended Sleep	*P-value (SS vs. ES)*
**Bed Time**	2338 (3h30m)	2330 (2h10m)	0.72	2223 (1h19m)	0.39
**TIB (hr)**	6.18 (0.38)	7.92 (0.52)	0.0001	8.42 (0.25)	0.0001
**TST (hr)**	5.92 (0.41)	7.53 (0.30)	0.0002	7.93 (0.35)	0.0002
**LTS (min)**	10.2 (5.2)	17.7 (7.5)	0.03	17.6 (7.1)	0.005
**SE (%)**	95.7 (2.0)	95.0 (2.5)	0.54.	94.2 (2.3)	0.12
**Number of Awakenings per night**	0.9 (0.6)	0.7 (0.5)	0.42	1.5 (1.7)	0.17
**Duration of Awakenings (min)**	5.1 (4.8)	6.1 (8.7)	0.71	14.3 (14.7)	0.15
**Naps (min)**	6.07 (9.4)	13.2 (2.5)	0.51	3.30 (4.7)	0.25
**Caffeine Intake (mg)**	252.8 (198.3)	85 (79)	0.04	257.4 (229.7)	0.85

TIB  =  time in bed, TST  =  total sleep time, LTS  =  latency to sleep, SE  =  sleep efficiency.

## Results

### Normal sleep vs. Short sleep

Neither group was significantly different in terms of subjective sleepiness (ESS [mean ± SD] 6.1±1.7 [NS] *vs*. 4.6±3.0 [SS], *t = 1.21; p = 0.24*) or insomnia (ISI 4.5±2.4 [NS] *vs*. 4.6±3.7 [SS], *t = −0.78; p = 0.98*).

#### Sleep diary

Table 1 presents the data from the sleep diary and indicates that habitual TST was significantly shorter in the SS group than in normal sleepers at baseline as required by group assignment (5 h 55 m ±0.4 *vs*. 7 h 32 m ±0.3; *t = 6.81, p<0.01*). Time in bed was significantly shorter as well in the SS (6 h 11 m ±0.38) compared to NS subjects (7h 55m ± 0.52) (*t = 6.76, p<0.01*). In addition, there was a longer latency to sleep onset in the NS group than in SS group (17.7 min *vs*. 10.2 min, *t = 2.29; p<0.04*) suggesting increased sleep drive associated with the short sleep duration.

There was no significant difference between groups in sleep efficiency, number and duration of awakenings, and frequency and duration of naps (Table 1). However, caffeine intake was significantly higher in the SS group than in NS subjects (means 252 vs. 85 mg, respectively; *t = −2.21; p<0.04*), providing further indication of habitual sleep restriction in this group.

#### P50/GDW in Normal sleep vs. Short sleep

**Figure 1:**
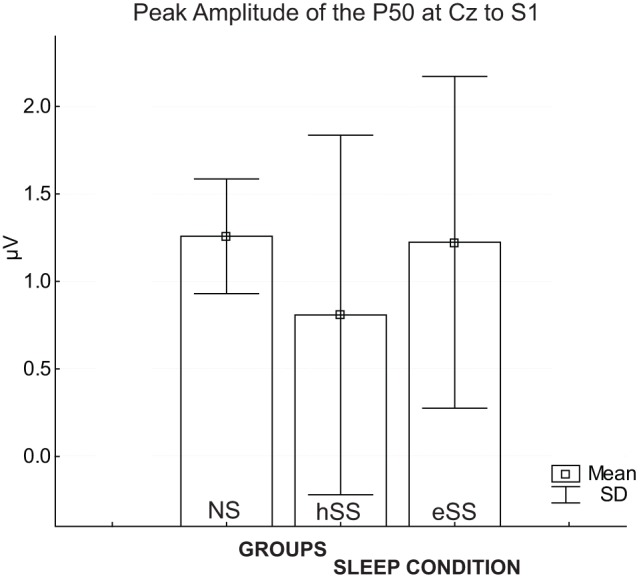
The means and SDs for peak amplitude of the P50 response at Cz electrode to click-sound S1 for each group (NS, SS) and sleep condition (hSS and eSS).

**Figure 2:**
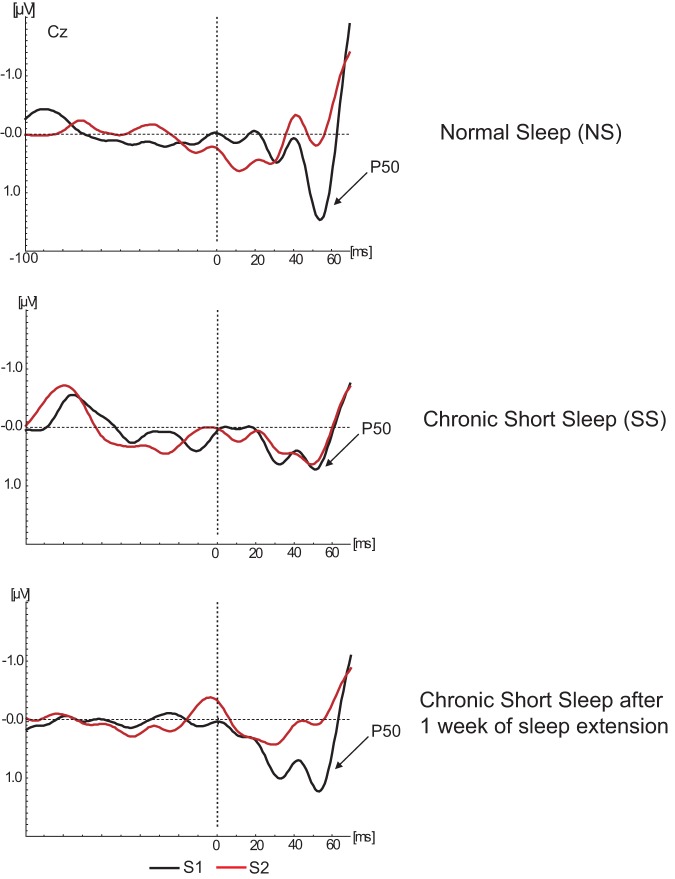
Grand average of the P50 waveforms corresponding to S1 (black line) and S2 (red line) obtained from normal sleeping (NS) and habitual short sleeping (hSS) individuals. Illustration of changes in P50 amplitude after one week of sleep extension in habitual short sleeping (eSS) individuals is on the bottom of the figure.

Figure 1 presents the means and SD of the peak amplitude of P50 responses elicited by S1 obtained from the NS and SS groups. Comparison between groups revealed that the peak amplitude of P50 to S1 in NS ([mean ± SD] 1.36 µV ±0.8 µV) was not significantly different from that in the SS group (0.8 µV ±1.0 µV) (*F(1.13) = 1.36; p = 0.23*). Figure 2 illustrates the grand averages of P50 ERP waveforms to S1 and S2 in NS and SS groups.

**Figure 3:**
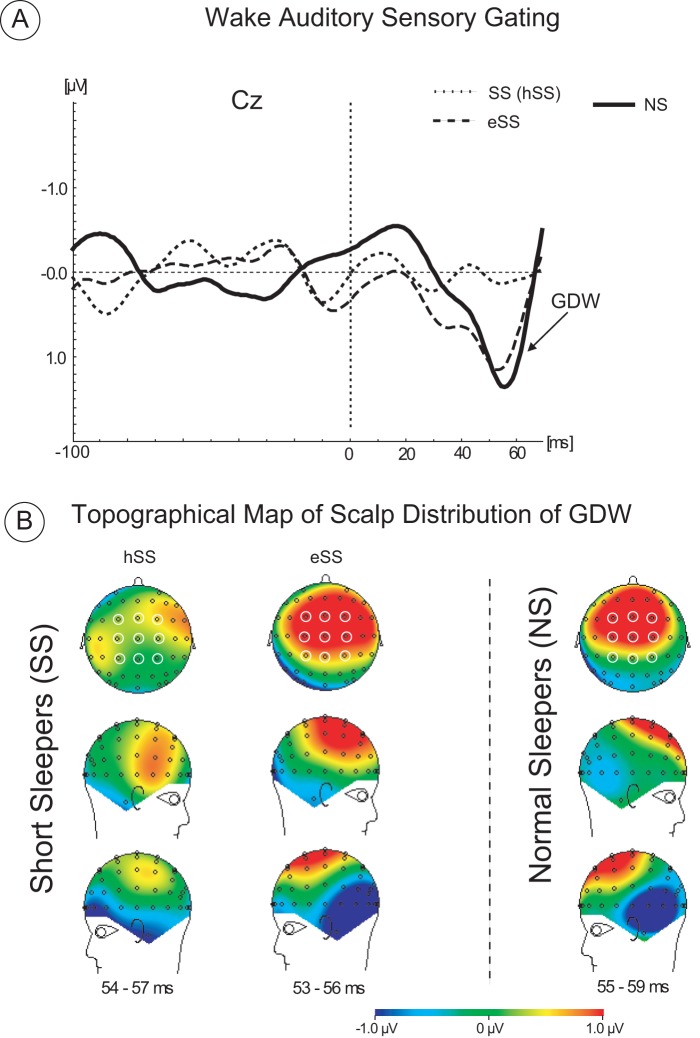
Group average difference waveforms corresponding to gating difference waveform (GDW) obtained from normal sleepers (NS), habitual short sleeping subjects (SS) after their habitual sleep (hSS) and extended sleep (eSS). (B) Topographic scalp distribution of GDW for each group and sleep condition. The open circles on the maps indicate the electrodes selected for comparison of the GDW amplitude and statistical evaluation. The selected time latencies corresponding to the largest peak of the averaged GDW waveforms calculated for each group and sleep condition. The red color indicates the positive polarity and the blue color indicates the negative polarity of the GDW waveform.

As illustrated in Figure 3A, the GDW showed lower mean amplitude in the SS group compared to that of the NS group. The analysis of the main effect of Group [mean ± SD]: 0.32 µV ±0.55 µV [SS] vs. 0.8 µV ±0.10 µV [NS] *F(1.13)  = 6.34; p<0.02*) confirmed that GDW in SS was significantly lower relative to that in NS when all 9 electrodes were combined. There were no other significant main effects.

The results of the group × laterality interaction (*F(2.28)  = 4.68; p<0.02*) showed that the reduction of GDW amplitude in the SS group was due to a lower amplitude of GDW over the left hemisphere and over central-midline sites relative to that in the NS group ([F3, C3, and P3] 0.01 µV vs. 0.6 µV, respectively: post hoc test *p<0.05*) and ([Fz, Cz, and Pz] 0.3 µV vs. 1.2 µV, respectively; *p<0.01*), (see Figure 3B, topographical map of scalp distribution of GDW). There were no other significant interactions observed.

### Habitual Short Sleep vs. Extended Sleep

#### Sleep diary

Table 1 summarizes the sleep diary data obtained from subjects after their habitual sleep and a week of sleep extension. There were significant differences between sleep conditions for time in bed as required by the protocol (6 h 11 m ±0.38 [hSS] *vs.* 8 h 25 m ±0.25 [eSS], *t = −16.2; p<0.01*). In addition to that, total sleep time was significantly increased (5 h 55 m ±0.41 [hSS] *vs*. 7 h 32 m ±0.30 [eSS], *t = −13.9; p<0.01*), as was latency to sleep onset (10.2 m ±5.2 m [hSS] *vs.* 17.6 m ±7.1 m [eSS]; *t = −4.01; p<0.03*; (see Table 1)). Objective confirmation of the differences in total sleep time recorded on the diary for the SS was made using a newly validated portable EEG sleep monitoring device [39]. Specifically, SS obtained 5.6 h ±0.55 min of total sleep time during their week of habitual sleep and 7.9 h ±0.35 min of total sleep time during their week of extended sleep (*t = −2.26; p<0.001*). The number and duration of awakenings increased in the extended TIB condition, but were not significantly different between the two sleep conditions (see Table 1).

#### P50/GDW in Habitual Short Sleep *vs*. Extended Sleep

As illustrated in Figures 1 and 2, the P50 response to S1 was not significantly different between sleep conditions in habitual short sleepers at the Cz electrode (0.8 µV ±0.4 µV [TST  = 5 h 55 m] *vs.* 1.2 µV ±0.9 µV [TST  = 7 h 32 m), *(F(1.7) = 0.84; p = 0.3*)), suggesting that perception of the first click for both conditions was similar.

Figure 3A shows the increase in amplitude of the GDW after sleep extension relative to the habitual short sleep condition (1.03 µV ±0.5 µV *vs*. 0.33 µV ±0.2 µV, respectively). The analysis of the main effect of the TIB conditions confirmed that this difference is statistically significant (*F(1,7) = 15.989, p<0.005*) when all 9 electrodes were combined. There were no other significant main effects observed.

The sleep condition × frontality interaction analysis confirmed that sleep extension significantly increased the amplitude of GDW over frontal and central brain areas (0.9 µV [eSS] *vs*. 0.2 µV [hSS] across F3, Fz and F4; *p<0.002*; and 1.2 µV [eSS] *vs*. 0.3 µV [hSS] across C3, Cz and C4, *p<0.001*), with a minimal and non-significant difference in GDW amplitude over parietal areas between sleep conditions. Figure 3B illustrates the voltage map of differences for the GDW corresponding to hSS and eSS sleep conditions. The greatest difference in GDW mean amplitude distribution (coded by red) is present over left frontal and central areas between sleep conditions. However, there is a minimal difference between the topographical scalp distribution of GDW corresponding to eSS and NS. No other interactions were significantly different.

#### P50/GDW in Short sleep after 1 week of sleep extension *vs*. Normal sleep

In order to evaluate the differences between normal sleepers *vs*. short sleepers after sleep extension for the peak amplitude of the P50 response and corresponding GDW, the following comparisons were performed. The peak of the P50 to S1 in normal sleepers was not significantly different from the peak of the P50 to S1 in short sleepers after sleep extension [1.3 µV ±0.8 µV vs. 1.2 µV ±0.9 µV, *p = 0.6)*, see Figure 1.

Analysis of the main effect of group did not reveal significant differences between mean amplitude of the GDW corresponding to normal sleepers (1.2 µV when all 9 electrodes were combined) and short sleepers after sleep extension (0.9 µV when all 9 electrodes were combined) *(F (1,14) = 1.29; p = 0.3)*.

The analysis of the main effect of laterality revealed significant differences for the smaller amplitude of the GDW over left and the right hemisphere (collapsed across F3, C3 and P3[left] and across F4, C4 and P4[right] electrodes) compared to the GDW measured at central electrodes (collapsed Fz, Cz and Pz) *(F(2,28) = 3.66; p<0.04)*. The mean amplitude of GDW between left and right hemispheres was not statistically different (0.6 µV [left hemisphere] *vs*. 0.8 µV [right hemisphere]) when the two groups were combined.

There was no statistical significance observed for the analysis of the main effect for frontality or any interactions.

## Discussion

The present study provides new evidence for waking auditory sensory gating deficits in habitual short sleepers who appear to be sleep restricted. Among insomnia subjects, relative to normally sleeping individuals, research has demonstrated reduced gating only during *wakefulness*, but not during stage 2 and REM sleep [22]. Our finding, consistent with study of Milner and colleagues, supports the fact that habitual short sleep (∼6 hrs/night) negatively affects pre-attentive processes during wakefulness, probably due to an increased level of homeostatic sleep pressure even in individuals who do not report their sleepiness. It is important to recognize that many patients with disorders of daytime sleepiness deny being sleepy. The same is true for chronic insufficient sleep in the absence of a primary sleep disorder [40].

A higher level of homeostatic sleep pressure measured by theta/low alpha frequencies during wakefulness was demonstrated in *short sleepers* compared to *long sleepers* (TST >9 h) in a study by Aeschbach and colleagues [41]. The reduction of gating found in our habitual short sleepers may be related to increased homeostatic sleep pressure. These data also show that chronic short sleepers had higher caffeine consumption relative to normal sleeping subjects. However, the reduced gating may suggest the presence of a sleep deficit and associated neurophysiological deficit in the habitual short sleepers. It is possible that habitual short sleepers use caffeine as an alertness-enhancing agent to behaviorally regulate their basal sleepiness which they may not recognized [7], [40]. The reduced amplitude of the gating in habitual short sleepers in our study is objective evidence of a neuronal deficit associated with short total sleep time. Additionally, it was previously found that caffeine is able to modulate the amplitude of the auditory sensory gating in healthy volunteers [32].

The present results show that one week of extended time in bed led to increased total sleep time from 5.92 h to 7.93 h in habitual short sleepers as assayed by a sleep diary. Although only 8 subjects per groups were used in the present study, the GDW effect sizes assessed here were generally quite robust (effect sizes of 0.50 (NS vs. hSS) can be considered as “moderate,” and 0.90 (hSS vs. eSS) as “strong”; see [42]). The amplitude of the gating was increased from 0.3 µV to 1 µV, in habitual short sleepers which was similar to the amplitude of the gating in normal sleeping subjects. Maximum enhancement of the gating amplitude after extended sleep was observed over frontal and central electrodes compared to parietal electrodes, suggesting a positive effect of sleep extension on fronto–central brain regions. Moreover, the largest reduction of the gating amplitude was observed over the left hemisphere and central areas in habitual short sleepers compared to that of normal sleepers, suggesting a higher vulnerability of the left hemisphere to sleep loss. Based on previous research, it is known that the gating response is primarily mediated by the auditory cortex and prefrontal cortex [25], [26]. Previous magnetoencephalography (MEG) studies indicate that the M50 (P50 EEG) component from the left auditory cortex is predictive of both psychophysical and neuropsychological measures of preattentional and attentional functioning and may be more useful at distinguishing between healthy and clinical populations [43]–[45]. In these studies, the reduced gating ratio derived from the left auditory cortex M50 component is correlated with lower P50 EEG gating and with impaired neuropsychological measures of attentional functioning [44]. However, none of these correlational relationships existed for the right auditory cortex. These results suggest that there may be an increased left hemispheric specialization for sensory gating processes which is impacted by habitual sleep restriction. In our study, the habitual short sleepers demonstrated a reduction of gating over the left hemisphere but not the right hemisphere. Importantly, sleep extension improved gating in habitual short sleepers to the level of normal sleeping individuals, although “complete” normalization of the entire network may require more than one week of sleep extension. It was shown that two weeks of sleep extension was associated with alertness improvement in normal subjects [1], [46] more effectively as compared to the alertness improvement following one week of extended sleep.

What is the functional significance of reduced waking sensory gating? Functionally, attention is impacted by sleep loss, and the gradual decline of attention measured by prolonged reaction time associated with the build-up of homeostatic sleep pressure has been proposed as a *behavioral biomarker of sleepiness* [40]. The neurophysiological brain mechanisms of changes in the distributed attentional network from a state of *alertness* to a state of *sleepiness* will require further investigation. It has been hypothesized, however, that the origin of this gradual increase in reaction time and attentional lapses in response to sleep restriction might be explained by momentary shifts (probably by inhibitory mechanisms) between centers of the sleep (e.g., hypothalamus, thalamus and brainstem) [47] and wakefulness systems [48], [49] resulting in “state instability” [48]; for review see also [50]. Interestingly, the disappearance of P50 during slow wave sleep and reappearance of this component again during REM and wakefulness suggests that, functionally, at least some of the neuronal generators of P50 are related to arousal state [51], [52]. Thus, the importance of P50-gating studies on individuals with reduced sleep time is that we can determine the neuronal changes associated with sleep reduction that may lead to dysregulation of the sleep–wake cycle and potentially result in attentional deficits. Since the GDW is computed as a difference between brain activity in response to S1 and S2, therefore it reflects the difference in the brain activity during processing of S1 and S2 stimuli. The GDW is different between groups presumably because the network underlying GDW is impacted by sleep loss in one group and normally functioning in normal sleepers. A previously published study suggested that neuronal activity underlying the GDW might be located within frontal lobe circuitry [25], [26]. Therefore, it is reasonable to hypothesize that the difference in GDW between groups is related to differences in frontal lobe neuronal activity. Obviously, further study of source localization of P50 gating is needed to evaluate the brain locations related to GDW in sleep deprived individuals. The association between sleep duration and the amplitude of sensory gating suggests that the measurement of GDW is a potentially useful *brain biomarker of homeostatic sleep pressure*. This possibility awaits further investigation using objective measures of homeostatic sleep pressure and sleepiness such as the multiple sleep latency test and spectral analyses of slow frequency EEG bands, especially in pathological populations with increased sleep drive.

## Conclusion

The primary comparison showed evidence of impaired auditory sensory gating in habitual short sleeping individuals, measured by P50 ERP. Neurophysiologically, habitual sleep restriction is associated with the inability to filter out extraneous sensory information. However, extended sleep may reverse this effect and normalize the amplitude of the auditory sensory gating process in healthy subjects during wakefulness.

## References

[1] Roehrs T, Timms V, Zwyghuizen-Doorenbos A, Roth T (1989) Sleep extension in sleepy and alert normals. Sleep 12: 449–457. 2799218 10.1093/sleep/12.5.449

[2] Cote KA, Milner CE, Osip SL, Baker ML, Cuthbert BP (2008) Physiological arousal and attention during a week of continuous sleep restriction. Physiol Behav 95: 353–364. 18655799 10.1016/j.physbeh.2008.06.016

[3] Gumenyuk V, Roth T, Korzyukov O, Jefferson C, Bowyer S, et al (2011) Habitual short sleep impacts frontal switch mechanism in attention to novelty. Sleep 34: 1659–1670. 22131603 10.5665/sleep.1430 PMC3208843

[4] Knutson KL, Van Cauter E, Rathouz PJ, DeLeire T, Lauderdale DS (2010) Trends in the prevalence of short sleepers in the USA: 1975–2006. Sleep 33: 37–45. 20120619 10.1093/sleep/33.1.37 PMC2802246

[5] Dinges DF, Pack F, Williams K, Gillen KA, Powell JW, et al (1997) Cumulative sleepiness, mood disturbance, and psychomotor vigilance performance decrements during a week of sleep restricted to 4–5 hours per night. Sleep 20: 267–277. 9231952

[6] Aeschbach D, Cajochen C, Landolt H, Borbely AA (1996) Homeostatic sleep regulation in habitual short sleepers and long sleepers. Am J Physiol 270: R41–53. 8769783 10.1152/ajpregu.1996.270.1.R41

[7] Kamdar BB, Kaplan KA, Kezirian EJ, Dement WC (2004) The impact of extended sleep on daytime alertness, vigilance, and mood. Sleep Med 5: 441–448. 15341888 10.1016/j.sleep.2004.05.003

[8] Cajochen C, Brunner DP, Krauchi K, Graw P, Wirz-Justice A (2000) EEG and subjective sleepiness during extended wakefulness in seasonal affective disorder: circadian and homeostatic influences. Biol Psychiatry 47: 610–617. 10745053 10.1016/s0006-3223(99)00242-5

[9] Gosselin A, De Koninck J, Campbell KB (2005) Total sleep deprivation and novelty processing: implications for frontal lobe functioning. Clin Neurophysiol 116: 211–222. 15589199 10.1016/j.clinph.2004.07.033

[10] Thomas M, Sing H, Belenky G, Holcomb H, Mayberg H, et al (2000) Neural basis of alertness and cognitive performance impairments during sleepiness. I. Effects of 24 h of sleep deprivation on waking human regional brain activity. J Sleep Res 9: 335–352. 11123521 10.1046/j.1365-2869.2000.00225.x

[11] Drummond SP, Bischoff-Grethe A, Dinges DF, Ayalon L, Mednick SC, et al (2005) The neural basis of the psychomotor vigilance task. Sleep 28: 1059–1068. 16268374

[12] Lijffijt M, Lane SD, Meier SL, Boutros NN, Burroughs S, et al (2009) P50, N100, and P200 sensory gating: relationships with behavioral inhibition, attention, and working memory. Psychophysiology 46: 1059–1068. 19515106 10.1111/j.1469-8986.2009.00845.x PMC2821570

[13] McGhie A, Chapman J (1961) Disorders of attention and perception in early schizophrenia. Br J Med Psychol 34: 103–116. 13773940 10.1111/j.2044-8341.1961.tb00936.x

[14] Adler LE, Pachtman E, Franks RD, Pecevich M, Waldo MC, et al (1982) Neurophysiological evidence for a defect in neuronal mechanisms involved in sensory gating in schizophrenia. Biol Psychiatry 17: 639–654. 7104417

[15] Clementz BA, Geyer MA, Braff DL (1997) P50 suppression among schizophrenia and normal comparison subjects: a methodological analysis. Biol Psychiatry 41: 1035–1044. 9129784 10.1016/S0006-3223(96)00208-9

[16] Boutros NN, Belger A (1999) Midlatency evoked potentials attenuation and augmentation reflect different aspects of sensory gating. Biol Psychiatry 45: 917–922. 10202580 10.1016/s0006-3223(98)00253-4

[17] Freedman R, Adler LE, Waldo MC, Pachtman E, Franks RD (1983) Neurophysiological evidence for a defect in inhibitory pathways in schizophrenia: comparison of medicated and drug-free patients. Biol Psychiatry 18: 537–551. 6134559

[18] Buchwald JS, Erwin R, Van Lancker D, Guthrie D, Schwafel J, et al (1992) Midlatency auditory evoked responses: P1 abnormalities in adult autistic subjects. Electroencephalogr Clin Neurophysiol 84: 164–171. 1372231 10.1016/0168-5597(92)90021-3

[19] Teo C, Rasco L, Skinner RD, Garcia-Rill E (1998) Disinhibition of the sleep state-dependent p1 potential in Parkinson's disease-improvement after pallidotomy. Sleep Res Online 1: 62–70. 11382858

[20] Uc EY, Skinner RD, Rodnitzky RL, Garcia-Rill E (2003) The midlatency auditory evoked potential P50 is abnormal in Huntington's disease. J Neurol Sci 212: 1–5. 12809992 10.1016/s0022-510x(03)00082-0

[21] Hairston IS, Talbot LS, Eidelman P, Gruber J, Harvey AG (2010) Sensory gating in primary insomnia. Eur J Neurosci 31: 2112–2121. 20529120 10.1111/j.1460-9568.2010.07237.x

[22] Milner CE, Cuthbert BP, Kertesz RS, Cote KA (2009) Sensory gating impairments in poor sleepers during presleep wakefulness. Neuroreport 20: 331–336. 19444955 10.1097/wnr.0b013e328323284e

[23] Hansotia P, Wall R, Berendes J (1985) Sleep disturbances and severity of Huntington's disease. Neurology 35: 1672–1674. 2932657 10.1212/wnl.35.11.1672

[24] Wiegand M, Moller AA, Lauer CJ, Stolz S, Schreiber W, et al (1991) Nocturnal sleep in Huntington's disease. J Neurol 238: 203–208. 1832711 10.1007/BF00314781

[25] Korzyukov O, Pflieger ME, Wagner M, Bowyer SM, Rosburg T, et al (2007) Generators of the intracranial P50 response in auditory sensory gating. Neuroimage 35: 814–826. 17293126 10.1016/j.neuroimage.2006.12.011 PMC1993359

[26] Mayer AR, Hanlon FM, Franco AR, Teshiba TM, Thoma RJ, et al (2009) The neural networks underlying auditory sensory gating. Neuroimage 44: 182–189. 18801443 10.1016/j.neuroimage.2008.08.025 PMC2656944

[27] Buchwald JS, Rubinstein EH, Schwafel J, Strandburg RJ (1991) Midlatency auditory evoked responses: differential effects of a cholinergic agonist and antagonist. Electroencephalogr Clin Neurophysiol 80: 303–309. 1713841 10.1016/0168-5597(91)90114-d

[28] Garcia-Rill E (1997) Disorders of the reticular activating system. Med Hypotheses 49: 379–387. 9421802 10.1016/s0306-9877(97)90083-9

[29] Steriade M, Datta S, Pare D, Oakson G, Curro Dossi RC (1990) Neuronal activities in brain-stem cholinergic nuclei related to tonic activation processes in thalamocortical systems. J Neurosci 10: 2541–2559. 2388079 10.1523/JNEUROSCI.10-08-02541.1990 PMC6570275

[30] Ucar E, Lehtinen EK, Glenthoj BY, Oranje B (2012) The effect of acute exogenous melatonin on P50 suppression in healthy male volunteers stratified for low and high gating levels. J Psychopharmacol 26: 1113–1118. 22331175 10.1177/0269881111430752

[31] Ghisolfi ES, Prokopiuk AS, Becker J, Ehlers JA, Belmonte-de-Abreu P, et al (2002) The adenosine antagonist theophylline impairs p50 auditory sensory gating in normal subjects. Neuropsychopharmacology 27: 629–637. 12377399 10.1016/S0893-133X(02)00337-8

[32] Ghisolfi ES, Schuch A, Strimitzer IM Jr, Luersen G, Martins FF, et al (2006) Caffeine modulates P50 auditory sensory gating in healthy subjects. Eur Neuropsychopharmacol 16: 204–210. 16278075 10.1016/j.euroneuro.2005.09.001

[33] Belenky G, Wesensten NJ, Thorne DR, Thomas ML, Sing HC, et al (2003) Patterns of performance degradation and restoration during sleep restriction and subsequent recovery: a sleep dose-response study. J Sleep Res 12: 1–12. 12603781 10.1046/j.1365-2869.2003.00337.x

[34] Johns MW (2000) Sensitivity and specificity of the multiple sleep latency test (MSLT), the maintenance of wakefulness test and the epworth sleepiness scale: failure of the MSLT as a gold standard. J Sleep Res 9: 5–11. 10733683 10.1046/j.1365-2869.2000.00177.x

[35] Bastien CH, Vallieres A, Morin CM (2001) Validation of the Insomnia Severity Index as an outcome measure for insomnia research. Sleep Med 2: 297–307. 11438246 10.1016/s1389-9457(00)00065-4

[36] Netzer NC, Stoohs RA, Netzer CM, Clark K, Strohl KP (1999) Using the Berlin Questionnaire to identify patients at risk for the sleep apnea syndrome. Ann Intern Med 131: 485–491. 10507956 10.7326/0003-4819-131-7-199910050-00002

[37] McNair DM, Lorr M., Droppleman L.F. (1992) Profile of mood states (POMS) manual.

[38] Zouridakis G, Boutros NN (1992) Stimulus parameter effects on the P50 evoked response. Biol Psychiatry 32: 839–841. 1450298 10.1016/0006-3223(92)90088-h

[39] Shambroom JR, Fabregas SE, Johnstone J (2012) Validation of an automated wireless system to monitor sleep in healthy adults. J Sleep Res 21: 221–230. 21859438 10.1111/j.1365-2869.2011.00944.x

[40] Balkin TJ (2011) Behavioral biomarkers of sleepiness. J Clin Sleep Med 7: S12–15. 22003322 10.5664/JCSM.1344 PMC3190422

[41] Aeschbach D, Postolache TT, Sher L, Matthews JR, Jackson MA, et al (2001) Evidence from the waking electroencephalogram that short sleepers live under higher homeostatic sleep pressure than long sleepers. Neuroscience 102: 493–502. 11226688 10.1016/s0306-4522(00)00518-2

[42] Cohen J (1988) Statistical power analysis for the behavioral sciences. Hillsdale, N.J.: L. Erlbaum Associates. xxi, 567 p.

[43] Hanlon FM, Miller GA, Thoma RJ, Irwin J, Jones A, et al (2005) Distinct M50 and M100 auditory gating deficits in schizophrenia. Psychophysiology 42: 417–427. 16008770 10.1111/j.1469-8986.2005.00299.x

[44] Thoma RJ, Hanlon FM, Moses SN, Edgar JC, Huang M, et al (2003) Lateralization of auditory sensory gating and neuropsychological dysfunction in schizophrenia. Am J Psychiatry 160: 1595–1605. 12944333 10.1176/appi.ajp.160.9.1595

[45] Thoma RJ, Hanlon FM, Moses SN, Ricker D, Huang M, et al (2005) M50 sensory gating predicts negative symptoms in schizophrenia. Schizophr Res 73: 311–318. 15653276 10.1016/j.schres.2004.07.001

[46] Roehrs T, Shore E, Papineau K, Rosenthal L, Roth T (1996) A two-week sleep extension in sleepy normals. Sleep 19: 576–582. 8899937

[47] Saper CB, Chou TC, Scammell TE (2001) The sleep switch: hypothalamic control of sleep and wakefulness. Trends Neurosci 24: 726–731. 11718878 10.1016/s0166-2236(00)02002-6

[48] Doran SM, Van Dongen HP, Dinges DF (2001) Sustained attention performance during sleep deprivation: evidence of state instability. Arch Ital Biol 139: 253–267. 11330205

[49] Durmer JS, Dinges DF (2005) Neurocognitive consequences of sleep deprivation. Semin Neurol 25: 117–129. 15798944 10.1055/s-2005-867080

[50] Banks S, Dinges DF (2007) Behavioral and physiological consequences of sleep restriction. J Clin Sleep Med 3: 519–528. 17803017 PMC1978335

[51] Erwin R, Buchwald JS (1986) Midlatency auditory evoked responses: differential effects of sleep in the human. Electroencephalogr Clin Neurophysiol 65: 383–392. 2427329 10.1016/0168-5597(86)90017-1

[52] Kisley MA, Olincy A, Freedman R (2001) The effect of state on sensory gating: comparison of waking, REM and non-REM sleep. Clin Neurophysiol 112: 1154–1165. 11516727 10.1016/s1388-2457(01)00578-8

